# Effects of Inspiratory Muscle Training on Respiratory Muscle Strength, Lactate Accumulation and Exercise Tolerance in Amateur Runners: A Randomized Controlled Trial

**DOI:** 10.3390/life15050705

**Published:** 2025-04-27

**Authors:** Zhe Ren, Junxia Guo, Yurong He, Yu Luo, Hao Wu

**Affiliations:** 1 School of Kinesiology and Health, Capital University of Physical Education and Sports, Beijing 100191, China; renzhe0208@163.com (Z.R.); guojunxia2022@cupes.edu.cn (J.G.); heyurong2022@cupes.edu.cn (Y.H.); luoyu2021@cupes.edu.cn (Y.L.); 2 Comprehensive Key Laboratory of Sports Ability Evaluation and Research of the General Administration of Sport of China, Beijing 100191, China; 3 Beijing Key Laboratory of Sports Function Assessment and Technical Analysis, Beijing 100191, China; 4 School of Rehabilitation, Gannan Medical University, Ganzhou 341000, China

**Keywords:** inspiratory muscle training, exercise tolerance, respiratory muscle strength, lactate accumulation, amateur runners

## Abstract

Objective: This study investigated the dose–response relationship of inspiratory muscle training (IMT) on respiratory muscle strength, lactic acid accumulation and exercise tolerance in amateur runners. Methods: Thirty male amateur runners were randomly assigned to three groups: a high-intensity IMT (HIMT) group, a low-intensity IMT (LIMT) group, and a control group. In addition to their regular training regimen, the high-intensity and low-intensity IMT groups underwent a supervised IMT protocol for a duration of 8 weeks. The primary outcome measures included maximal inspiratory pressure (MIP), maximal expiratory pressure (MEP), time to exhaustion (TTE), blood lactate (BLa), rate of perceived exertion (RPE), and rate of perceived breathlessness (RPB). Secondary outcomes encompassed VO2 max, forced vital capacity (FVC), forced expiratory volume in one second (FEV_1_), and the FEV_1_/FVC ratio. Results: After 8 weeks of IMT, the MIP of HIMT and LIMT were significantly improved (*p* < 0.01), and the MEP of both groups also increased (*p* < 0.01). There were no significant changes in FVC and FEV_1_ (*p* > 0.05), but only FEV_1_/FVC in HIMT was significantly improved (*p* < 0.01). Exercise testing showed a significant increase in TTE in both the HIMT and low LIMT groups (*p* < 0.01). Post-exercise RPE scores were lower in both the HIMT group (*p* < 0.01) and LIMT group (*p* < 0.05), and both HIMT and LIMT groups’ post-exercise RPB scores were also reduced in both (*p* < 0.05). In addition, blood lactate accumulation was significantly lower in both HIMT (*p* < 0.01) and LIMT (*p* < 0.05). There were no significant changes in VO2 max (*p* > 0.05) and HR peak (*p* > 0.05). Conclusion: IMT for 8 weeks can improve respiratory muscle strength, prolong exercise time, improve blood lactate accumulation, subjective fatigue, and dyspnea during exercise. Among them, high-intensity IMT can better improve exercise tolerance.

## 1. Introduction

The increasing emphasis on health and athleticism has led to a notable rise in participation in endurance activities, such as long-distance running and marathon events [1]. As global interest in recreational sports continues to grow, amateur runners face distinct physiological challenges. These challenges stem from their diverse training backgrounds and relatively lower baseline fitness levels compared to professional athletes [2]. Exercise tolerance is a crucial determinant of an athlete’s stamina, speed, and overall performance in aerobic exercises. As a result, improving exercise tolerance has become a key focus for runners [3,4,5]. However, traditional training paradigms frequently prioritize the development of limb muscles while overlooking the adaptation of respiratory muscles. This oversight is particularly concerning given emerging evidence suggesting that respiratory fatigue may serve as a limiting factor in endurance performance [6,7].

During exercise, a phenomenon known as “inspiratory muscle blood steal” can compromise the function of respiratory muscles, thereby limiting their capacity to oxygenate active skeletal muscles. This limitation may exacerbate fatigue in skeletal muscles and reduce exercise endurance [8]. Recent advancements in exercise physiology have emphasized the diaphragm’s dual role as both a respiratory pump and a contributor to postural stability during running, highlighting its potential as a target for performance optimization [9]. Vital to the regulation of blood-gas homeostasis during exercise, the ventilatory pump muscles work in unison to co-ordinate changes in pleural pressure, inspiratory and expiratory flow, lung volume, and aeration. The significance of respiratory muscle fatigue in this context is often underestimated. Respiratory muscle fatigue can significantly limit exercise performance. As physical exertion intensifies, the diaphragm and other respiratory muscles become fatigued, leading to a reduction in their efficiency and an increase in the perceived effort of breathing. This phenomenon is particularly detrimental during endurance activities, where maintaining a balance between oxygen delivery and muscular demand is critical [10]. Inspiratory muscle training (IMT) has been demonstrated to delay the onset of respiratory muscle fatigue during physical activity. IMT enhances athletic performance by reducing the rate of perceived exertion (RPE) and the rate of perceived breathlessness (RPB), while also modulating metabolic reflexes that regulate blood flow redistribution between respiratory and skeletal muscles [11,12]. Notably, IMT may influence lactate kinetics by improving the efficiency of respiratory muscles, thus delaying the accumulation of blood lactate—a key biomarker associated with anaerobic threshold and fatigue [13]. While existing meta-analyses support the benefits of IMT in elite athletes [14], its translational potential for amateur populations remains insufficiently explored. Recent studies on IMT have shown promising results in enhancing the performance of endurance athletes and physically active individuals. However, the effectiveness of IMT continues to be a subject of ongoing debate within the scientific community. To date, the literature has predominantly focused on the effects of IMT among professional athletes, while data concerning amateur runners are notably limited. This gap is significant, as amateur runners represent the largest demographic in endurance sports yet lack evidence-based guidelines tailored to their physiological profiles. Moreover, previous research has seldom incorporated multidimensional assessments of respiratory strength, lactate dynamics, and subjective exertion metrics—an integrative approach that is essential for elucidating the mechanistic pathways underlying IMT’s effects.

In response to the identified research gap, this study systematically investigated the effects of IMT on respiratory muscle strength, blood lactate levels, and exercise tolerance in amateur runners. Our research innovatively compares high-intensity versus low-intensity IMT protocols, addressing a significant controversy in training prescription. Assessments were conducted at baseline and after an 8-week IMT regimen. This study elucidates the dose–response relationships between IMT intensity and physiological adaptations, aiming to identify an effective training method to enhance exercise endurance in amateur runners. This study innovatively compares high- vs. low-intensity IMT protocols, addressing a critical gap in training prescription for amateur populations. The findings may have implications for reducing injury risk and promoting long-term adherence to endurance sports.

## 2. Materials and Methods

### 2.1. Participants

G*Power 3.1 software was utilized for sample size estimation, and repeated measures analysis of variance (ANOVA) was employed to assess the interaction effects between groups and time. The parameters incorporated into the calculation included an effect size (f) of 0.25, a significance level (α) of 0.05, and a statistical power of 80%. Considering a dropout rate of 10%, we determined that the required total sample size would be 30 participants [15]. Thirty male amateur runners (ages: 18–25 years) were recruited, all possessing at least three years of consistent running training experience. Baseline characteristics—including age, height, weight, BMI, and running experience were homogeneous across groups (Table 1).

Inclusion criteria: male, aged 18–25 years; at least three years of training experience; no history of cardiovascular, respiratory, or musculoskeletal disorders; participants were asked to be able to follow the study’s intervention and testing protocols. Exclusion criteria: subjects with chronic respiratory or cardiovascular disease (such as asthma, chronic obstructive pulmonary disease) or other contraindications; individuals with musculoskeletal injuries; and individuals who had other training prior to or during the study were deemed ineligible.

The study adhered to the principles of the Declaration of Helsinki. Ethical approval was obtained from the Research Ethics Committee of Capital University of Physical Education and Sport (Approval No. 2022A58), and written informed consent was secured from all participants prior to enrollment.

### 2.2. Experimental Design

This study investigated the effects of inspiratory muscle training (IMT) on amateur runners. The participants were divided into three groups: a high-intensity inspiratory muscle training group (HIMT group, 80% MIP, n = 10), a low-intensity inspiratory muscle training group (LIMT group, 50% MIP, n = 10), and a control group (CON group, n = 10). Over the course of 8 weeks, individuals in the IMT groups engaged in a supervised regimen five times per week, utilizing a progressive flow-resistance load breathing trainer. Each training session comprised two sets of 30 maximal inspiratory efforts. In contrast, the control group continued their regular running training without any additional inspiratory muscle training interventions. Randomization was conducted utilizing a computer-generated random number list to ensure an unbiased assignment of participants to groups (www.randomizer.org, accessed on 12 October 2022). Participants were allocated to their respective groups according to the established randomization sequence, and this allocation remained concealed until the completion of baseline assessments.

Baseline assessments were conducted at the onset of the study to establish initial values for respiratory muscle strength (MIP/MEP), pulmonary function (spirometry: FVC, FEV_1_/FVC), and exercise tolerance (time to exhaustion on a cycle ergometer with gas exchange analysis). Post-intervention evaluations mirrored baseline measures and were performed by blinded assessors under standardized laboratory conditions (22 °C, 50% humidity) at consistent circadian times (±1 h). Secondary outcomes included blood lactate levels, perceptual exertion assessed through Borg RPE/RPB scales, and aerobic capacity determined via VO_2_ max. Throughout the trial, participants maintained their regular running schedules while adhering to standardized pre-test hydration and dietary protocols verified through 24 h recalls. Additionally, they refrained from engaging in strenuous activities for 48 h prior to testing.

### 2.3. Respiratory Muscle Strength Test

Respiratory muscle strength was quantified via assessments of both inspiratory and expiratory muscle capacities. Inspiratory muscle strength was gauged by recording the MIP, whereas MEP was employed to evaluate the strength of the expiratory muscles [13]. According to the standards of the American Thoracic Society and European Respiratory Society, an RMT device (XeeK BW05, Xiamen, China) was utilized for both pre-intervention and post-intervention assessments. Using this device to measure MIP and MEP has been effective and reliable [16].

For the assessment of inspiratory muscle strength, subjects were required to be in a state of maximal expiration with a nose clip applied to prevent nasal air leakage, followed by a forceful and rapid inhalation. Expiratory muscle strength was determined by having subjects perform a maximal expiration from their normal breathing amplitude [17]. Subjects were instructed to exhale as quickly and forcefully as possible from a state of maximal inhalation. Each subject completed three attempts for both inspiratory and expiratory measurements, ensuring that the variability among trials did not exceed 5%. The highest values recorded were adopted as the definitive MIP and MEP values. All measurements were conducted with subjects in a standing posture.

### 2.4. Pulmonary Function Test

Before and following the intervention, participants underwent a battery of pulmonary function tests utilizing an intelligent respiratory training device (Xeek BW05, Xiamen, China). During the testing protocol, participants were instructed to adopt an erect posture, breathe through a suction mouthpiece, and utilize a nose clip, thereby ensuring the accuracy of the measurement data [18].

The pulmonary function tests were administered in alignment with the protocols delineated by the American Thoracic Society (1995). These tests included measuring forced vital capacity (FVC), forced expiratory volume in one second (FEV_1_), and the FEV_1_/FVC ratio. The variation among the trio of test attempts was constrained to within 5%, ensuring the selection of the most accurate test value to affirm the reliability and consistency of the data.

### 2.5. Exercise Test

Participants were subjected to an incremental exercise test on a cycle ergometer (Ergoline 100, Erlangen, Germany) during both pre- and post-8-week training intervention. After a 5 min warm-up at a low intensity, the test commenced with an initial workload of 50 W, which was escalated by increments of 10 W every minute. The cadence was maintained at a consistent 60 revolutions per minute until volitional exhaustion [19].

Before the commencement of the test, the demographic and physiological baseline data of the subjects were documented. After these preparations, a respiratory mask was fitted to each subject, and the cycle ergometer seat height was adjusted for optimal ergonomics. Throughout the exercise test, a CORTEX gas metabolism analyzer (MAX-II) was employed to continuously monitor expired gases and assess maximal oxygen uptake. Upon completion of the incremental exercise test, participants’ perceived exertion and breathlessness were evaluated using Borg’s rating of perceived exertion (RPE) and rate of perceived breathlessness (RPB) scales. The RPB scale is used in clinical and exercise physiology research [20,21]. Concurrently, heart rate (HR) was continuously recorded via the Polar V800 (Polar Electro OY; Kempele, Finland). Peak heart rate was recorded immediately after the exercise test. At rest and during peak exercise, blood lactate (BLa) levels were measured immediately using a portable lactate analyzer (EKF Lactate Scout 4, Magdeburg, Germany). Lactate samples were obtained from the earlobe, and the analyzer provides an instantaneous reading of lactate concentration.

The predetermined cessation criteria for the exercise test were multifaceted, including (1) achievement of 90% of the age-predicted maximum heart rate (HR max), calculated as 220 minus the subject’s age; (2) a respiratory quotient (RQ) surpassing 1.10; (3) the participant’s inability to maintain the required pedaling cadence, as evidenced by a Borg RPE score exceeding 17; and (4) a discernible plateau in oxygen uptake as depicted by the VO2 curve [22].

### 2.6. Inspiratory Muscle Training

Subjects are acclimated to the laboratory environment and are briefly introduced to the exercise regimen relevant to the study. They were then introduced to the IMT program. IMT intervention was performed with the flow resistance load breathing training apparatus (Xeek BW05, Xiamen, China) [16,23]. During IMT, participants used disposable filters and nose clips to block nasal breathing, thereby isolating the inspiratory muscles [24]. The IMT regimen involved overcoming a predetermined inspiratory resistance while allowing unimpeded expiration. Inhalation is characterized by rapid and robust initial effort, transitioning to a slower, sustained phase, followed by a protracted exhalation [25]. Participants were assigned to two groups based on the intensity of the IMT program: the HIMT group underwent training at 80% of their MIP, whereas the LIMT group trained at 50% of their MIP. The selection of 50% MIP for the low-intensity IMT (LIMT) group was informed by prior research indicating that this intensity effectively trains respiratory muscles while minimizing the risk of overfatigue in recreational athletes. Additionally, utilizing 50% MIP offers a greater training load [14]. Both groups trained for 5 days per week, twice daily, 30 inhalation repetitions per session [26,27].

### 2.7. Data and Statistical Analysis

The Shapiro–Wilk test was conducted to assess the normality of the distribution of the investigated variables. Results are presented as mean ± standard deviation (M ± SD). Group differences in baseline characteristics were evaluated using one-way analysis of variance (ANOVA). Independent samples t-tests were employed to compare the general characteristics of the participants. For the analysis of respiratory muscle strength, pulmonary function, and exercise performance between groups at baseline and following an eight-week training period, a 3 × 2 (group × time) repeated measures ANOVA was utilized, with Bonferroni correction for multiple comparisons. Effect size was calculated to assess the magnitude of the differences between groups. Partial eta squared (η^2^) was used as the measure of effect size for the repeated measures analysis of variance (ANOVA). Effect sizes were interpreted according to conventional thresholds: small (η^2^ = 0.01), medium (η^2^ = 0.06), and large (η^2^ = 0.14). Statistical significance was set at *p* < 0.05. All statistical analyses were performed using SPSS software (version 27.0) and GraphPad Prism (version 9.5).

## 3. Results

All participants completed the eight-week intervention. As shown in the base information (Table 1), no significant differences were observed between the groups at baseline (*p* > 0.05).

### 3.1. Respiratory Muscle Strength

**Figure 1:**
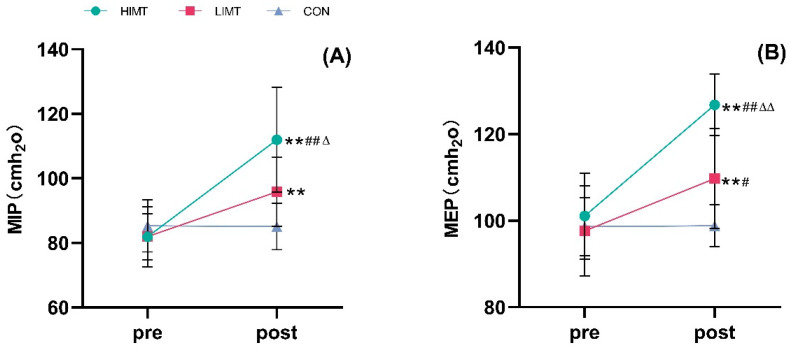
(**A**) MIP (Maximal inspiratory pressure) and (**B**) MEP (Maximal expiratory Pressure) pre- and post-intervention (Mean ± SD). ^#^ significant differences between HIMT and LIMT groups versus CON group post-intervention (*p* < 0.05), ^Δ^ significant difference between HIMT and LIMT groups post-intervention (*p* < 0.05), Compared with before training, ** *p* < 0.01; HIMT or LIMT compared with the control group, ^##^ *p* < 0.01; HIMT compared with LIMT, ^ΔΔ^ *p* < 0.01.

For MIP (Figure 1A), a significant main effect of time was observed (F = 102.64, *p* < 0.001, η^2^ partial = 0.792), along with a significant interaction between group and time (F = 36.796, *p* < 0.001, η^2^ partial = 0.732). After the 8-week intervention, MIP was significantly improved in both the HIMT and LIMT groups (*p* < 0.001). Notably, MIP was significantly higher in the HIMT group compared to both the LIMT group (*p* = 0.016) and the control group (*p* < 0.01).

Regarding MEP (Figure 1B), a significant main effect of time was found (F = 122.932, *p* < 0.001, η^2^ partial = 0.820), and the interaction between group and time was also significant (F = 41.669, *p* < 0.001, η^2^ partial = 0.755). MEP increased significantly in both the HIMT and LIMT groups after the intervention (*p* < 0.01). Post-intervention, the HIMT group showed significantly greater MEP values compared to both the LIMT group (*p* < 0.01) and the control group (*p* < 0.01), while the difference between the LIMT group and the control group was not statistically significant (*p* = 0.20) (Table 2).

### 3.2. Pulmonary Function

No significant improvements were observed in FVC or FEV_1_ in any of the groups following the intervention. However, the FEV_1_/FVC ratio showed a significant main effect of time (F = 13.918, *p* = 0.001, η^2^ partial = 0.340), as well as a significant interaction between group and time (F = 5.057, *p* = 0.014, η^2^ partial = 0.272). Notably, only the HIMT group demonstrated a significant improvement in the FEV_1_/FVC ratio after the intervention (*p* < 0.01) (Table 2). 

### 3.3. VO2 Max Data

**Figure 2:**
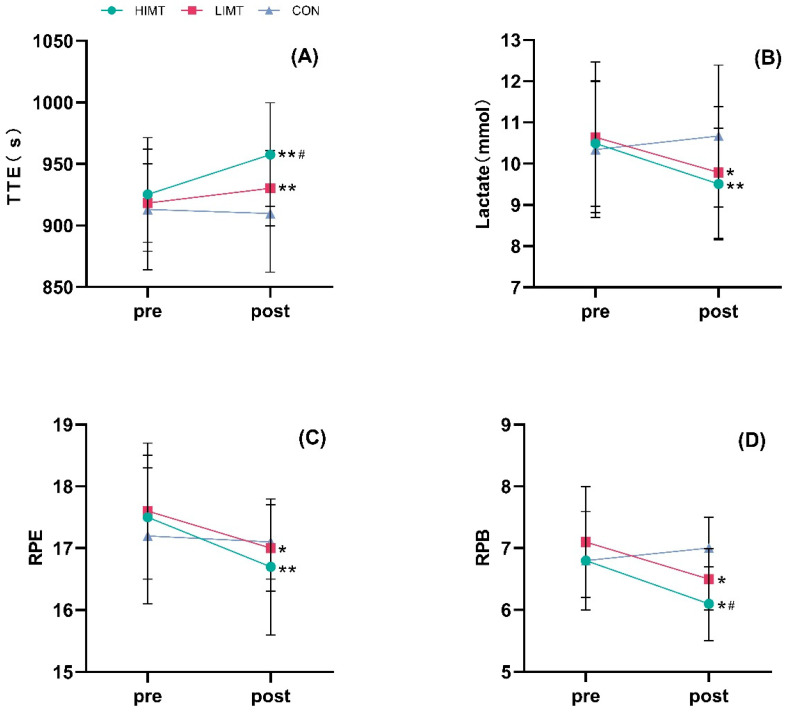
(**A**) TTE (time to exhaustion), (**B**) blood lactate concentration, (**C**) RPE (rate of perceived exertion), (**D**) RPB (rate of perceived breathlessness) pre and post-intervention (Mean ± SD). * Significant increase within-group post-intervention compared to pre-intervention (*p* < 0.05), ^#^ significant differences between HIMT and LIMT groups versus CON group post-intervention (*p* < 0.05), Compared with before training, ** *p* < 0.01.

No significant time effect was observed for VO2 max (F = 0.045, *p* = 0.834, η^2^ partial = 0.002), and the interaction between time and group was also not significant (F = 1.925, *p* = 0.165, η^2^ partial = 0.125). Neither VO2 max nor HR peak showed significant improvements in any of the groups following the 8-week intervention (*p* > 0.05). (Figure 2A) (Table 3).

### 3.4. Exercise Tolerance

For TTE (Figure 2B), a significant group × time interaction was observed (F = 23.067, *p* < 0.001, η^2^ partial = 0.631), with both the HIMT (*p* < 0.01) and LIMT (*p* = 0.025) groups showing a significant increase in exercise duration. Post-intervention, the difference in TTE between the HIMT and CON groups was also significant (*p* = 0.042).

Regarding RPE (Figure 2C), no significant group × time interaction was found (F = 1.857, *p* = 0.176, η^2^ partial = 0.121). However, post-exercise RPE was significantly reduced in both the HIMT (*p* = 0.005) and LIMT (*p* = 0.032) groups. For RPB (Figure 2D), there was a significant time × group interaction on RPB (F = 3.630, *p* = 0.040, η^2^ partial = 0.212). After the 8-week IMT intervention, RPB was significantly decreased immediately after exercise in HIMT group (*p* = 0.012) and LIMT group (*p* = 0.018).

Finally, there was no significant impact on the HR peak immediately following exercise, regardless of whether the assessment occurred before or after the intervention (*p* > 0.05). There was a significant group × time effect of BLA production during exercise (F = 5.251, *p* = 0.012, η^2^ partial = 0.280), and both HIMT (*p* = 0.004) and LIMT (*p* < 0.012) groups were significantly decreased after the 8-week intervention (Table 3).

## 4. Discussion

The primary outcomes of this investigation reveal that an 8-week regimen of IMT significantly increases respiratory muscle strength and markedly enhances exercise tolerance. This enhancement is evidenced by prolonged time to exhaustion, reduced lactate accumulation, and improved subjective experience. However, the intervention did not result in significant changes in VO2 max or endurance capacity. These findings suggest that IMT may potentially improve running performance in the studied cohort.

The observed improvements in respiratory muscle strength align with the physiological principle of specificity of training, consistent with the literature suggesting a 4–12 week timeframe for IMT to be efficacious [14]. Our research identified significant enhancements following an eight-week IMT course with intensities prescribed at 40% and 80% of MIP. Upon completion of both high- and low-intensity IMT regimens, participants exhibited marked improvements in MIP and MEP, with the high-intensity protocol more effectively augmenting respiratory muscle strength in amateur runners (*p* < 0.01). Notably, MIP increased by 36.7%, and MEP by 25.4%. These gains likely reflect neuromuscular adaptations, such as improved motor unit recruitment and synchronization, rather than structural hypertrophy, given the relatively short intervention period.

The diaphragm, the principal muscle responsible for inspiration, accounts for approximately 90% of ventilatory action and contributes to 65–70% of total respiratory muscle function. Morphologically and functionally similar to skeletal muscles, respiratory muscles respond to training stimuli in a manner comparable to other skeletal muscle groups [28]. High-load strength training principles dictate that maximal muscle strength enhancement requires substantial resistance to provoke a strong central nervous response [29], thereby increasing motor unit recruitment and synchronous muscle contraction for optimal physiological adaptations. These principles are equally applicable to inspiratory muscle training, with peak strength or power output achievable at 80% MIP [30]. The superiority of high-intensity IMT in this study supports the concept of overload specificity, where higher resistance induces greater neural drive and subsequent strength gains. Moreover, the muscle stretch reflex theory posits that increased inspiratory muscle strength can augment expiratory muscle strength through improved neuromuscular recruitment [31]. These theoretical underpinnings are substantiated by Kyeongbong’s research, corroborating our conclusion that targeted inspiratory muscle training can amplify respiratory muscle thickness and fortify strength [32]. This study also found that LIMT at 40% MIP significantly improved MIP and MEP. According to the force-velocity relationship, LIMT facilitated faster muscle contraction velocities against lower resistances, which translated into the observed increases in MIP and MEP within the LIMT cohort. Archiza’s work supports the notion that low-intensity training can enhance neuromuscular recruitment patterns, particularly in individuals naive to inspiratory muscle training, by improving breathing mechanics [27].

Over the 8-week training period, no significant improvements in static pulmonary function parameters were observed across groups. This is likely due to the insufficient duration of the intervention to induce structural adaptations in lung compliance or alveolar-capillary diffusion efficiency [14]. However, the isolated enhancement of the FEV_1_/FVC ratio in the HIMT group (*p* < 0.01) reflect enhanced expiratory flow dynamics via reduced airway resistance or improved respiratory muscle coordination [33].

The lack of significant improvement in VO2 max observed in this study aligns with the physiological specificity of inspiratory muscle training (IMT), which primarily enhances respiratory muscle endurance without substantially impacting systemic oxygen transport mechanisms. This divergence likely results from the fact that IMT-induced adaptations are localized to the respiratory musculature, as evidenced by improved fatigue resistance, without inducing the cardiovascular system overload typically required for central hemodynamic improvements. The unaltered VO2 max values merit further attention, as VO2 max is closely dependent on the efficiency of integrated oxygen pathways, including pulmonary diffusion, circulatory delivery, and mitochondrial oxidative phosphorylation capacity [30]. The findings of this study indicate that neither high-intensity nor low-intensity IMT, when applied over an 8-week period, resulted in significant improvements in VO2 max. It is important to consider genetic predisposition as a key factor influencing VO2 max enhancement, as it plays a significant role in the adaptive response to long-term training [19]. Furthermore, VO2 max is directly influenced by the intensity of physical activity and is dependent on factors such as capillary density within muscle tissue, as well as the quantity, size, and activity of mitochondrial enzymes in the cells of major muscle groups [34].

IMT devices have been explored as supplementary tools in sports training across various disciplines, including swimming, rowing, cycling, running, and soccer [35]. As exercise intensity increases, the proportion of total oxygen consumption by respiratory muscles rises from 3 to 5% at rest to 13–16% during exertion. This heightened demand during physical activity places additional stress on the pulmonary system’s oxygen uptake. Intensified respiratory muscle activity can lead to fatigue, characterized by increased oxygen consumption and blood flow to these muscles, which may compete with the blood flow required by skeletal muscles [33]. However, the blood flow may be insufficient to meet both the oxygen demands and to clear the lactic acid produced in the respiratory muscles, leading to its accumulation. The resulting buildup of lactic acid activates chemoreceptors, which in turn reduces the contractile function of the respiratory muscles, potentially causing respiratory muscle fatigue and a subsequent decrease in exercise tolerance [36].

Incorporating respiratory muscle strength training into exercise regimens has been shown to significantly enhance exercise tolerance [37,38,39]. Rożek-Piechura et al. reported that IMT improved respiratory mechanics in long-distance runners, resulting in better pulmonary ventilation and a reduced oxygen cost during exertion. These improvements were accompanied by lower blood lactate concentrations and increased exercise tolerance [39]. Similarly, Chang et al. found that a 4-week IMT program significantly enhanced inspiratory muscle strength, improved 800 m running performance, and reduced the rate of change in limb blood flow post-exercise [40].

This study revealed a significant 32.3 s extension in exercise duration among recreational runners following high-intensity IMT (*p* < 0.01), compared to a 12 s improvement with low-intensity IMT. Concurrently, reductions in RPE and RPB reached statistical significance (*p* < 0.05), corroborating prior research findings. The high-intensity intervention demonstrated superior efficacy, reducing subjective fatigue and dyspnea scores by 38% and 42%, respectively, compared to the low-intensity group. The observed improvement in exercise capacity following IMT can be attributed to the enhancement of respiratory muscle strength, which mitigates the sensation of dyspnea and enhances blood flow to skeletal muscles. This dual effect delays both lactic acid accumulation and the onset of fatigue in active musculature, thereby prolonging exercise duration [13].

The current study revealed a significant reduction in post-exercise lactate concentration following an eight-week IMT program during progressive load exercise, with high-intensity IMT resulting in a 9.3% decrease and low-intensity IMT leading to a 7.9% decrease. These findings align with those of Leddy et al., who reported that four weeks of IMT diminished lactate production in runners during a 4 km time trial [41]. Similarly, Brown et al. found that IMT can mitigate the increase in lactate levels experienced during the respiratory patterns of intense exercise [42]. The decrease in lactate production is likely due to improved lactate uptake and metabolism by the respiratory muscles post-IMT [43].

Despite efforts to be rigorous in the design and conduct of this study, some limitations need to be considered. First, the 8-week intervention period may not be sufficient to enable lung function or VO2 max, measures that typically require longer training to show significant adaptation. Second, inspiratory muscle fatigue during exercise testing was not assessed before and after IMT in this study.

Future studies should aim to increase demographic diversity by including participants with varying fitness levels and exercise backgrounds to enhance the generalizability of the findings. Additionally, mechanistic studies incorporating techniques such as diaphragmatic electromyography or real-time flow imaging could provide deeper insights into the underlying mechanisms by which IMT modulates respiratory and motor interactions, as well as its effects on metabolic reflexes. These approaches could help clarify the precise physiological pathways through which IMT improves respiratory muscle function and exercise performance.

It is recommended that amateur runners add IMT to their regular running training to improve respiratory muscle strength, exercise tolerance, and reduce the risk of early fatigue during competition or prolonged training. Despite the positive results achieved with both high-intensity and low-intensity IMT protocols, even low-intensity training (50% MIP) can provide substantial benefits without inducing fatigue, making it a viable option for recreational athletes. By strengthening respiratory muscles, IMT helps delay the onset of fatigue, reduces perceived dyspnea during prolonged exercise, and improves overall endurance performance. In addition, since respiratory muscle fatigue is often overlooked in endurance training, integrating IMT can also shorten recovery time and reduce the possibility of overtraining, providing a non-pharmacological method to improve athletic performance.

## 5. Conclusions

Both high-intensity and low-intensity IMT for 8 weeks improved respiratory muscle strength, prolonged exercise time, and showed better improvements in blood lactate accumulation, subjective fatigue, and dyspnea during exercise. The effects of high-intensity IMT were significantly greater and provided better overall enhancement of exercise tolerance; IMT was not effective in improving spirometry and VO2 max, and future studies should explore the long-term effects and generalizability of IMT.

## Figures and Tables

**Table 1:** Descriptive statistics of essential information in each group (Mean ± SD).

Variable	HIMT	LIMT	Con	*p*
Age (yrs)	22.29 ± 1.25	22.57 ± 1.51	21.86 ± 1.77	0.684
Height (cm)	175.63 ± 4.67	177.86 ± 5.34	178.20 ± 4.97	0.589
Weight (kg)	67.79 ± 5.21	67.30 ± 5.04	65.41 ± 4.11	0.630
BMI (kg/m^2^)	21.9 ± 1.21	21.2 ± 1.14	20.5 ± 1.05	0.625
Running experience (yrs)	5.29 ± 1.11	5.43 ± 1.40	5.14 ± 1.35	0.918
HR (bpm)	61.80 ± 3.74	61.50 ± 4.40	60.70 ± 4.30	0.830
LA (mmol)	1.73 ± 0.40	2.07 ± 0.63	1.90 ± 0.48	0.354

**Table 2:** Changes in pulmonary function and respiratory muscle strength and post-intervention (Mean ± SD).

Variable	Time	HIMT	LIMT	Con
MIP (cmH_2_o)	Pre	81.90 ± 9.33	81.97 ± 7.12	85.27 ± 8.08
	Post	111.99 ± 16.21 **^##Δ^	95.86 ± 10.69 **	85.10 ± 7.21
MEP (cmH_2_o)	Pre	101.12 ± 9.95	97.64 ± 10.47	98.64 ± 6.75
	Post	126.78 ± 7.12 **^##ΔΔ^	109.74 ± 11.57 **^#^	98.82 ± 4.84
FVC (mL)	Pre	3770.2 ± 306.7	3842.4 ± 452.7	3788.0 ± 264.0
	Post	3800.6 ± 306.0	3839.1 ± 378.2	3472.0 ± 947.2
FEV_1_ (mL)	Pre	2856.0 ± 228.0	2937.8 ± 341.7	2885.4 ± 256.3
	Post	2963.6 ± 244.7	2955.6 ± 296.6	2647.2 ± 727.4
FEV_1_/FVC	Pre	0.75 ± 0.02	0.76 ± 0.03	0.76 ± 0.02
	Post	0.78 ± 0.02 **	0.77 ± 0.02	0.76 ± 0.03

^#^ significant differences between HIMT and LIMT groups versus CON group post-intervention (*p* < 0.05); Compared with before training, ** *p* < 0.01; HIMT or LIMT compared with the control group, ^##^ *p* < 0.01; HIMT compared with LIMT, ^Δ^ *p* < 0.05, ^ΔΔ^ *p* < 0.01.

**Table 3:** Changes in exercise test data pre- and post-intervention (Mean ± SD).

Variable	Time	HIMT	LIMT	Con
VO2 max (ml/kg/min)	Pre	44.60 ± 1.58	45.00 ± 2.61	44.80 ± 1.48
	Post	45.00 ± 1.83	44.70 ± 1.77	44.60 ± 1.51
Power (W)	Pre	204.3 ± 7.7	202.9 ± 5.26	202.1 ± 8.05
	Post	209.6 ± 7.06 **^#^	204.5 ± 5.13 *	201.4 ± 7.86
HR peak (bpm)	Pre	175.30 ± 6.95	176.40 ± 7.09	175.60 ± 8.76
	Post	173.90 ± 6.40	175.10 ± 6.01	176.00 ± 7.63
TTE (s)	Pre	925.30 ± 46.25	918.23 ± 31.90	913.06 ± 49.00
	Post	957.55 ± 42.18 **^#^	930.25 ± 30.55 **	909.78 ± 47.71
Lactate (mmol)	Pre	10.49 ± 1.53	10.64 ± 1.83	10.34 ± 1.65
	Post	9.51 ± 1.36 **	9.79 ± 1.60 *	10.67 ± 1.72
RPE	Pre	17.5 ± 1.0	17.6 ± 1.1	17.2 ± 1.1
	Post	16.7 ± 1.1 **	17.0 ± 0.7 *	17.1 ± 0.6
RPB	Pre	6.8 ± 0.8	7.1 ± 0.9	6.8 ± 0.8
	Post	6.1 ± 0.6 *^#^	6.5 ± 0.5 *	7.0 ± 0.5

^∗^ Significant increase within-group post-intervention compared to pre-intervention (*p* < 0.05); ^#^ significant differences between HIMT and LIMT groups versus CON group post-intervention (*p* < 0.05); Compared with before training, ** *p* < 0.01.

## Data Availability

The data that support the findings of this study are available on request from the corresponding author.
